# Therapeutic vs. Suprapharmacological Metformin Concentrations: Different Effects on Energy Metabolism and Mitochondrial Function in Skeletal Muscle Cells *in vitro*

**DOI:** 10.3389/fphar.2022.930308

**Published:** 2022-07-06

**Authors:** Kasja Pavlovic, Nina Krako Jakovljevic, Andjelka M. Isakovic, Tijana Ivanovic, Ivanka Markovic, Nebojsa M. Lalic

**Affiliations:** ^1^ Clinic for Endocrinology, Diabetes and Metabolic Diseases, University Clinical Center of Serbia, Belgrade, Serbia; ^2^ Faculty of Medicine, Institute of Medical and Clinical Biochemistry, University of Belgrade, Belgrade, Serbia; ^3^ Faculty of Medicine, University of Belgrade, Belgrade, Serbia

**Keywords:** metformin, mitochondria, skeletal muscle, therapeutic concentration, respirometry

## Abstract

Metformin is an oral antidiabetic agent that has been widely used in clinical practice for over 60 years, and is currently the most prescribed antidiabetic drug worldwide. However, the molecular mechanisms of metformin action in different tissues are still not completely understood. Although metformin-induced inhibition of mitochondrial respiratory chain Complex I and activation of AMP-activated protein kinase have been observed in many studies, published data is inconsistent. Furthermore, metformin concentrations used for *in vitro* studies and their pharmacological relevance are a common point of debate. The aim of this study was to explore the effects of different metformin concentrations on energy metabolism and activity of relevant signaling pathways in C2C12 muscle cells *in vitro*. In order to determine if therapeutic metformin concentrations have an effect on skeletal muscle cells, we used micromolar metformin concentrations (50 µM), and compared the effects with those of higher, millimolar concentrations (5 mM), that have already been established to affect mitochondrial function and AMPK activity. We conducted all experiments in conditions of high (25 mM) and low glucose (5.5 mM) concentration, in order to discern the role of glucose availability on metformin action. According to our results, micromolar metformin treatment did not cause Complex I inhibition nor AMPK activation. Also, cells cultured in low glucose medium were more sensitive to Complex I inhibition, mitochondrial membrane depolarization and AMPK activation by millimolar metformin, but cells cultured in high glucose medium were more prone to induction of ROS production. In conclusion, even though suprapharmacological metformin concentrations cause Complex I inhibition and AMPK activation in skeletal muscle cells *in vitro*, therapeutic concentrations cause no such effect. This raises the question if these mechanisms are relevant for therapeutic effects of metformin in skeletal muscle.

## Introduction

Metformin is an oral antidiabetic drug from the biguanide group. Relevant organizations recommend metformin as a first line drug for treating type 2 diabetes mellitus (American Diabetes Association, 2022). Due to its efficacy, favorable safety profile and affordable price, it is currently the most prescribed antidiabetic in the world (Diabetes Prevention Program Research Group, 2012). Metformin lowers blood glucose by inhibiting hepatic glucose production and increasing glucose uptake in peripheral tissues, mainly skeletal muscle (Inzucchi et al., 1998; Giannarelli et al., 2003; Phielix et al., 2011). Although it has been used in clinical practice for over 60 years, its molecular mechanisms of action are not yet fully elucidated.

Some of the commonly proposed mechanisms are inhibition of mitochondrial respiratory chain Complex I (CI) (El-Mir et al., 2000; Owen et al., 2000) and activation of AMP-activated protein kinase (AMPK) (Zhou et al., 2001; Musi et al., 2002). Clear conclusions about the precise mechanisms, target tissues and pharmacological relevance of these molecular effects of metformin are hard to draw, since published data is variable and inconsistent. Part of this variability can be explained by differences in chosen models, methods and experimental design. Also, there are significant differences in concentrations and durations of treatments that lead to observable effects. Concentrations of metformin used in *in vitro* studies are a common point of criticism (Fontaine, 2018; Vial et al., 2019), as most frequently reported concentrations are in the millimolar range, whereas plasma concentrations in conditions of oral administration rarely exceed 30 µM (Christensen et al., 2011). A small number of recent studies that used lower metformin concentrations showed either no change or an improvement of mitochondrial function (Wang et al., 2019; Emelyanova et al., 2021).

Metformin is also assumed to act as an insulin sensitizer, but it is unclear if this is caused by changes in insulin signaling at the cellular level, or changes in whole-body glucose and lipid metabolism (Zabielski et al., 2017; Bradley et al., 2019). Data on the direct effects of metformin on the phosphoinositide 3-kinase (PI3K)/Akt signaling pathway are inconsistent (Kim et al., 2002; Zhou et al., 2016), but having in mind the established relationship of this pathway and the AMPK pathway, which are regulated by one another at multiple sites (Towler and Hardie, 2007), it is safe to say that further exploration of this signaling axis is of importance for clarifying the full scope of metformin action. The insulin sensitizing effect of metformin could prove especially important for peripheral tissues like skeletal muscle, as previously discussed mechanisms have been studied and confirmed almost exclusively in liver cells. Studies on metformin action in skeletal muscle have been scarse in recent years, but as it is a tissue with a large impact in glycemic regulation, its potential role in glucose-lowering effects of metformin should not be disregarded.

Our hypothesis is that low metformin concentrations, in the therapeutic range, have a different effect on energy metabolism and activity of relevant signaling pathways in skeletal muscle cells than the previously described suprapharmacological concentrations. In order to determine this we used micromolar metformin concentrations (50 µM), to treat muscle cells *in vitro*, and we performed all experiments with high concentration (5 mM) in parallel, to see if the previously established effects would be reproduced in C2C12 cells and in our conditions. We conducted all experiments in conditions of high (25 mM) and low glucose (5.5 mM), as we consider energy substrate availability to be important, especially when studying an agent that is believed to alter pathways of glucose metabolism.

## Material and Methods

### Cell Culture and Reagents

C2C12 mouse myoblast cell line was obtained from European Collection of Animal Cell Cultures (ECACC). Cells were cultured at 37°C, in a humidified atmosphere with 5% CO_2_, in two different culture mediums—Dulbecco’s Modified Eagle Medium (DMEM) with high glucose (HG) (25 mM glucose) or low glucose (LG) (5.5 mM glucose), supplemented with 10% fetal bovine serum and 1% antibiotic/antimycotic solution (all from Capricorn Scientific, Ebsdorfergrund, Germany). Cells were prepared for experiments using the conventional trypsinization procedure with trypsin/EDTA, counted and plated in 96-well plates (3000 cells/well) for viability assays, 12-well plates (40,000 cells/well) for flow cytometry, Petri dishes (400,000 cells) for high-resolution respirometry and western blot (tissue culture plates were from Sarstedt, Numbrecht, Germany). After plating, cells were treated on the subsequent day with a solution of metformin hydrochloride (Galenika, Belgrade, Serbia) (concentration and duration of treatment are indicated in figure legends). If not otherwise stated cells were undifferentiated. For differentiation, cells were grown until reaching confluency, standard growth medium was altered to differentiation medium (DMEM supplemented with 2% horse serum), and cultured for another 4 days.

### Cell Viability

Cell viability was determined using three different viability assays - acid phosphatase (AcP), MTT and crystal violet (CV). For AcP assay, substrate solution (10 mM p-nitrophenyl phosphate in 0.1 M sodium acetate buffer, pH 5.5, with 0.1% Triton X-100) was added to cell culture. Cells were incubated for 90 min at 37°C, reaction was blocked by adding 1 M NaOH, and absorbance measured at 405 nm. For MTT assay, cell medium was removed after treatment and 3-(4,5-dimethylthiazol-2-yl)-2,5-diphenyltetrazolium bromide (MTT) solution (0.5 mg/ml) added to cell culture and incubated for 3 h at 37°C. The solution was discarded and formazan crystals solubilized by adding DMSO, and absorbance was measured at 570 nm. For CV assay, cell medium was discarded after the treatment and wells were washed with PBS, to remove the non-viable cells. The remaining cells were then fixed with methanol and stained with CV. The dye was diluted by adding 33% acetic acid and absorbance measured at 570 nm. For all viability assays, absorbance was measured using an automated microplate reader (Sunrise; Tecan, Dorset, United Kingdom). Viability of treated cells was expressed as a percentage of control (untreated) cells, which was considered to be 100%.

### Glucose Concentration

Glucose concentration in cell medium was measured as an indirect indicator of glucose uptake by the cells. Glucose concentration was measured spectrophotometrically, using Glucose-TR kit (Spinreact, Girona, Spain), which is based on an enzymatic reaction of glucose oxidation by glucose oxidase, followed by formation of hydrogen peroxide which reacts with a chromogenic oxygen acceptor, phenol-aminophenazone in the presence of peroxidase, and the intensity of formed color is proportional to the glucose concentration in the sample. Absorbance at 492 nm was measured using an automated microplate reader (Sunrise; Tecan, Dorset, United Kingdom). Each measurement was performed in duplicate, and mean values were used for further statistical analysis.

### Mitochondrial Respiration

Mitochondrial respiratory function was measured by high-resolution respirometry (Oroboros Oxygraph-2k, Oroboros Instruments, Innsbruck, Austria). After treatment, cells were trypsinized, counted and resuspended in mitochondrial respiration medium MiR05 (EGTA 0.5 mM, MgCl_2_⋅6H_2_O 3 mM, lactobionic acid 60 mM, taurine 20 mM, KH_2_PO_4_ 10 mM, HEPES 20 mM, D-sucrose 110 mM, BSA 1 g/l, pH 7.1), and experiments were performed in the same medium. For experiments on permeabilized cells, detergent digitonin was used to make pores in cell membranes, in order for substances that are not cell-permeable to be able to reach the mitochondria. Optimal cell number per chamber (500,000 cells) and digitonin concentration (10 µg/million cells) were determined according to protocol (Doerrier et al., 2018). Mitochondrial respiration was assessed using different substrate-uncoupler-inhibitor titration (SUIT) protocols, and expressed as O_2_ flow per cell (amol∙s-1∙cell-1). Respiration of permeabilized cells was assessed by measuring ROUTINE respiration of living (intact) cells prior to permeabilization by digitonin (10 µg/million cells) followed by LEAK respiration, which compensates for electron leak through the inner mitochondrial membrane, when ADP is not present and ATP synthase is not active, in presence of NADH-linked substrates (pyruvate 5 mM; malate 2 mM; glutamate 10 mM). NADH-linked oxidative phosphorylation capacity was measured in presence of NADH-linked substrates and ADP (ADP 2.5 mM; Mg 1.5 mM), and convergent NADH and succinate-linked oxidative phosphorylation capacity was measured after addition of succinate (10 mM). Succinate-linked oxidative phosphorylation capacity was obtained by inhibiting CI (rotenone 0.5 µM), ROX—residual oxygen consumption, after inhibition of Complex III (antimycin A 2.5 µM) and CIV—activity of Complex IV by addition of artificial Complex IV-specific electron donor (N,N,N′,N′-tetramethyl-p-phenylenediamine dihydrochloride - TMPD 0.5 mM; ascorbate 2 mM). All respiratory states presented in figures are corrected for ROX. Respiratory states measured and presented for experiments on living (intact) cells are ROUTINE - respiration of living cells, LEAK—respiration in presence of ATP synthase inhibitor oligomycin, ET—electron transfer capacity, i.e., respiration in presence of uncoupler (carbonyl cyanide m-chlorophenyl hydrazone—CCCP). Cytochrome *c* was used for assessing outer mitochondrial membrane integrity. For experiments performed in differentiated and undifferentiated cells with 5 days metformin treatment, citrate synthase activity, as a measure of mitochondrial content, was measured spectrophotometrically, according to protocol (Eigentler, 2020) modified for 96-well plate. All reagents for high-resolution respirometry were purchased from Sigma-Aldrich (St. Louis, Missouri, United States).

### Oxidative Stress and Mitochondrial Function

Cells were stained with dihydroethidium (DHE) for superoxide anion radical detection, dihydrorhodamine (DHR) for total production of reactive oxygen species (ROS), JC-1 for mitochondrial membrane potential and MitoTracker Red CMXRos for mitochondrial content (all from Thermo Fisher Scientific, Waltham, Massachusetts, United States). Cells were incubated with appropriate fluorochromes (1 µM DHR, 10 µM DHE, 2 μM JC-1, 100 nM MitoTracker Red) for 30 min. Fluorescence intensity was measured using FACSCalibur flow cytometer and analysed using CellQuest Pro Software (BD, Franklin Lakes, New Jersey, United States). Results for JC-1 are presented as green/red fluorescence ratio (FL1/FL2). All results are presented as fold change of GeoMean (geometric mean of detected fluorescence) compared to control (untreated) cells, set to 1.

### Signaling Pathway Activity

Signaling pathway activity was measured by western blot. For preparing samples, cells were lysed with buffer (15 mM Tris pH 7.4, 150 mM NaCl, 1% NP-40, 1 mM EDTA) with PMSF, phosphatase inhibitors NaF and Na_2_VO_3_ and protease/phosphatase inhibitor cocktail (Sigma-Aldrich; St Louis, Missouri, United States). After incubation (30 min on ice), samples were centrifuged at 14,000 g for 15 min at 4°C and supernatants were collected. Total protein concentration was measured by Bradford assay, and samples prepared by adding loading buffer (final concentrations in sample 80 mM Tris, 2% SDS, 10% glycerol, 2 mM β-mercaptoethanol, 0.9 mM bromophenol blue) and boiling at 100°C for 5 min. Equal amounts of sample (10 µg protein/lane) were separated by SDS-PAGE using 8–12% gels (depending on the proteins of interest). Proteins were transferred to nitrocellulose membranes (GE Healthcare, Chicago, Illinois, United States) by semi-dry transfer. Membranes were incubated with primary antibodies against proteins of interest: pAMPK Thr172 (#2535), AMPK (#2603), pAkt Ser473 (#9271), pAkt Thr308 (#4056), Akt (#9272), pGSK3β Ser9 (#5558), GSK3β (#12456), pACC Ser79 (#11818), ACC (#3676) (1:1000 dilution, all from Cell Signaling Technology; Danvers, Massachusetts, United States) and β-actin (PA5-85291) (1:10000 dilution, from Invitrogen, Thermo Fisher Scientific, Waltham, Massachusetts, United States) over night at 4°C and peroxidase-conjugated goat anti-rabbit IgG secondary antibody (111-035-144) (1:10,000 dilution, from Jackson Immuno Research Laboratories; West Grove, Pennsylvania, United States) for 90 min at room temperature. Chemiluminescent visualization was performed with luminol and p-coumaric acid solution using ChemiDoc. Protein levels were quantified by densitometry, using Image Lab software. Proteins of interest were normalized to β-actin, and phosphorylated forms were additionally normalized to the total levels of the same protein. Results are presented as fold change compared to untreated cells, set to 1. All equipment for SDS-PAGE, transfer and visualization, including software for densitometry is from Bio-Rad (Bio-Rad Laboratories Inc., Hercules, California, United States).

### Statistical Analysis

Student’s t-test was used for one factor comparison of two groups, and one way ANOVA for one factor comparison of three or more groups. Post hoc tests were performed according to Dunnett. A *p* value ≤0.05 was considered significant and ≤0.01 highly significant. Statistical tests were done using GraphPad Prism software (GraphPad Prism Software Inc., San Diego, California, United States).

## Results

**FIGURE 1:**
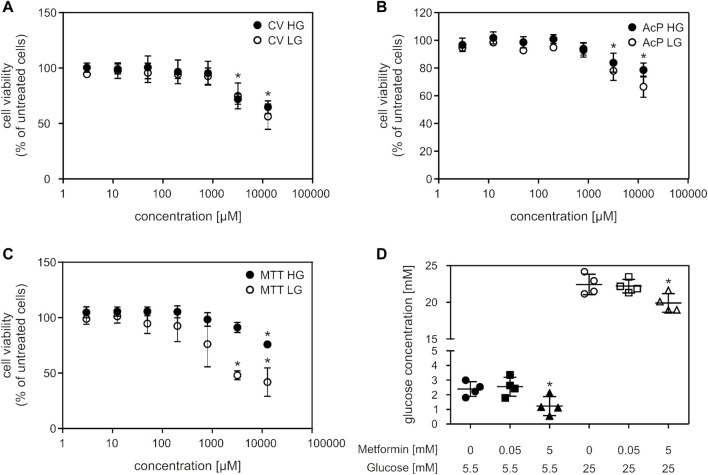
Millimolar metformin concentrations decreased cell viability and glucose concentration in cell medium. C2C12 cells were treated with a wide range of metformin concentrations (3; 12.5; 50; 200; 800; 3,200; 12,800 µM) for 24 h and cell viability was measured by CV **(A)**, AcP **(B)** and MTT **(C)** assays. Results from three independent experiments (each with six technical replicates) are presented as mean ± SD **(A–C)**. Glucose concentration in HG and LG cell medium after 24 h of treatment with 50 μM and 5 mM metformin was measured by Glucose-TR assay **(D)**. Results from four independent experiments are presented as scatter plots, with mean ± SD **(D)**, *p < 0.05. MF—metformin, CV—crystal violet, AcP—acid phosphatase, HG—high glucose, LG—low glucose.

Metformin treatment caused a decrease in cell viability in concentrations higher than 3 mM (3.2 and 12.8 mM), in two of the cell viability assays (CV and AcP) (Figures 1A,B). In the MTT assay, viability of cells grown in high glucose (HG) medium decreased only when treated with the highest metformin concentration (Figure 1C). High metformin concentration (5 mM) caused a decrease in glucose concentration in both HG and LG medium, where lower metformin concentration (50 µM) had no effect (Figure 1D).

**FIGURE 2:**
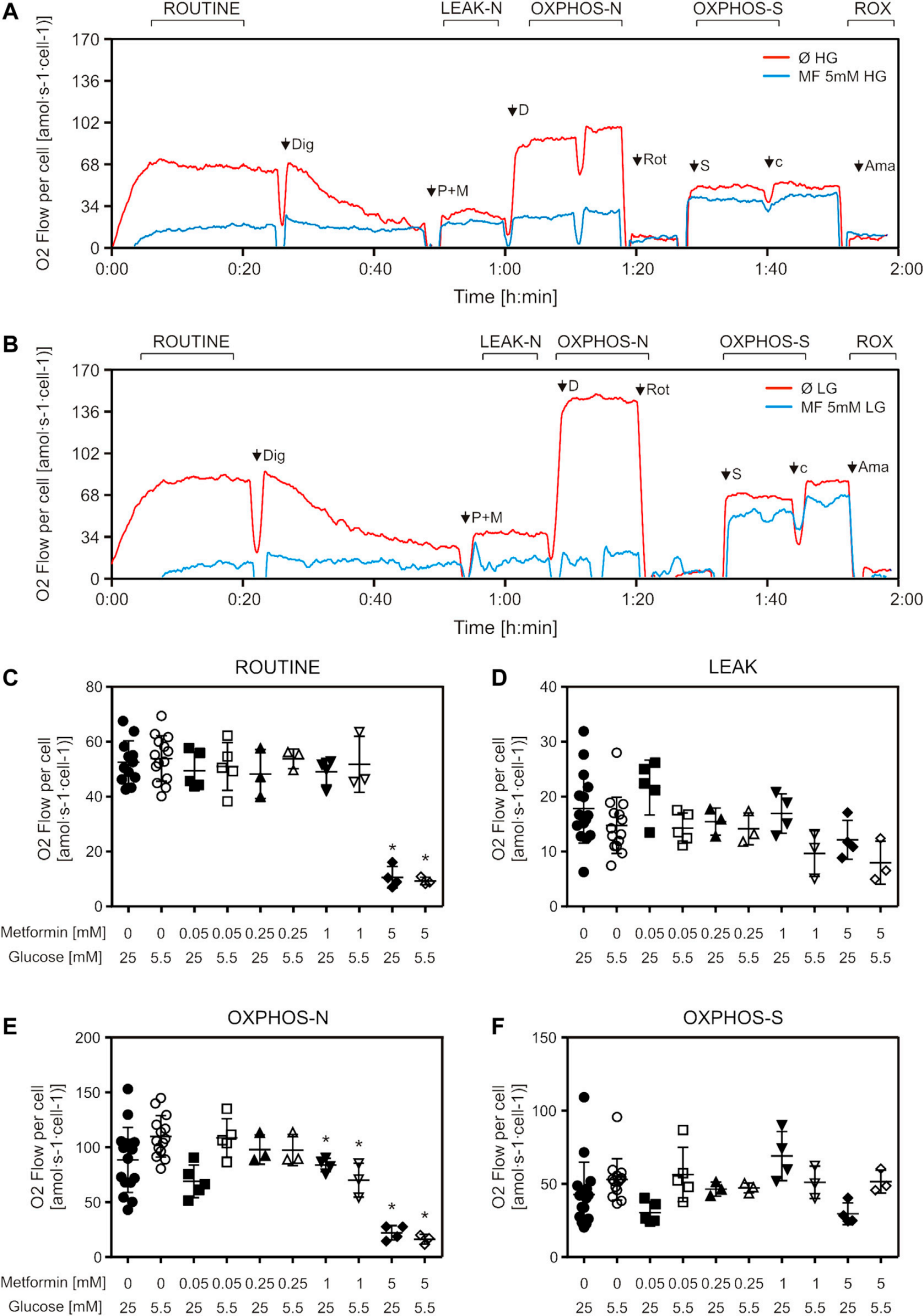
Mitochondrial respiration is decreased by suprapharmacological but not therapeutic metformin concentrations. C2C12 cells were treated with metformin (50–5000 µM) for 24 h and mitochondrial respiration of permeabilized cells was measured by high-resolution respirometry. Representative DatLab traces of untreated and 5 mM metformin treated high glucose **(A)** and low glucose **(B)** medium-cultured cells. Respiration rates of ROUTINE **(C)**, LEAK **(D)**, OXPHOS-N **(E)**, and OXPHOS-S **(F)** respiratory states, all corrected for ROX, from 3 to 5 independent experiments are presented as scatter plots, with mean ± SD, *p < 0.05. Dig—digitonin, P—pyruvate, M—malate, D—ADP, Rot—rotenone, S—succinate, cyt c—cytochrome *c*, Ama—antimycin A, HG—high glucose, LG—low glucose, MF—metformin, Ø—untreated control, OXPHOS-N—NADH-linked oxidative phosphorylation capacity, OXPHOS-S—succinate-linked oxidative phosphorylation capacity.

When we treated cells with increasing metformin concentrations for 24 h, we observed a decrease in ROUTINE respiration only in cells treated with the highest concentration (5 mM) (Figures 2A–C), and a decrease in oxidative phosphorylation capacity with NADH (Complex I)-linked substrates (OXPHOS-N) in cells treated with 1 and 5 mM metformin, in both cell media (Figures 2A,B,E). No differences were observed in either LEAK respiration or succinate (Complex II)-linked OXPHOS capacity (OXPHOS-S) (Figures 2D,F). Cells cultured in LG medium were more sensitive to metformin treatment—when treated with 5 mM metformin, the decrease in NADH-linked OXPHOS capacity was more pronounced (68% for HG and 85% for LG) (Figures 2A,B,E). OXPHOS capacity was higher in untreated LG, compared to HG medium-cultured cells (Figures 2A,B,E), which can be explained by the Warburg-Crabtree effect (Marroquin et al., 2007; de Kok et al., 2021), and was previously shown for C2C12 cells (Elkalaf et al., 2013).

**FIGURE 3:**
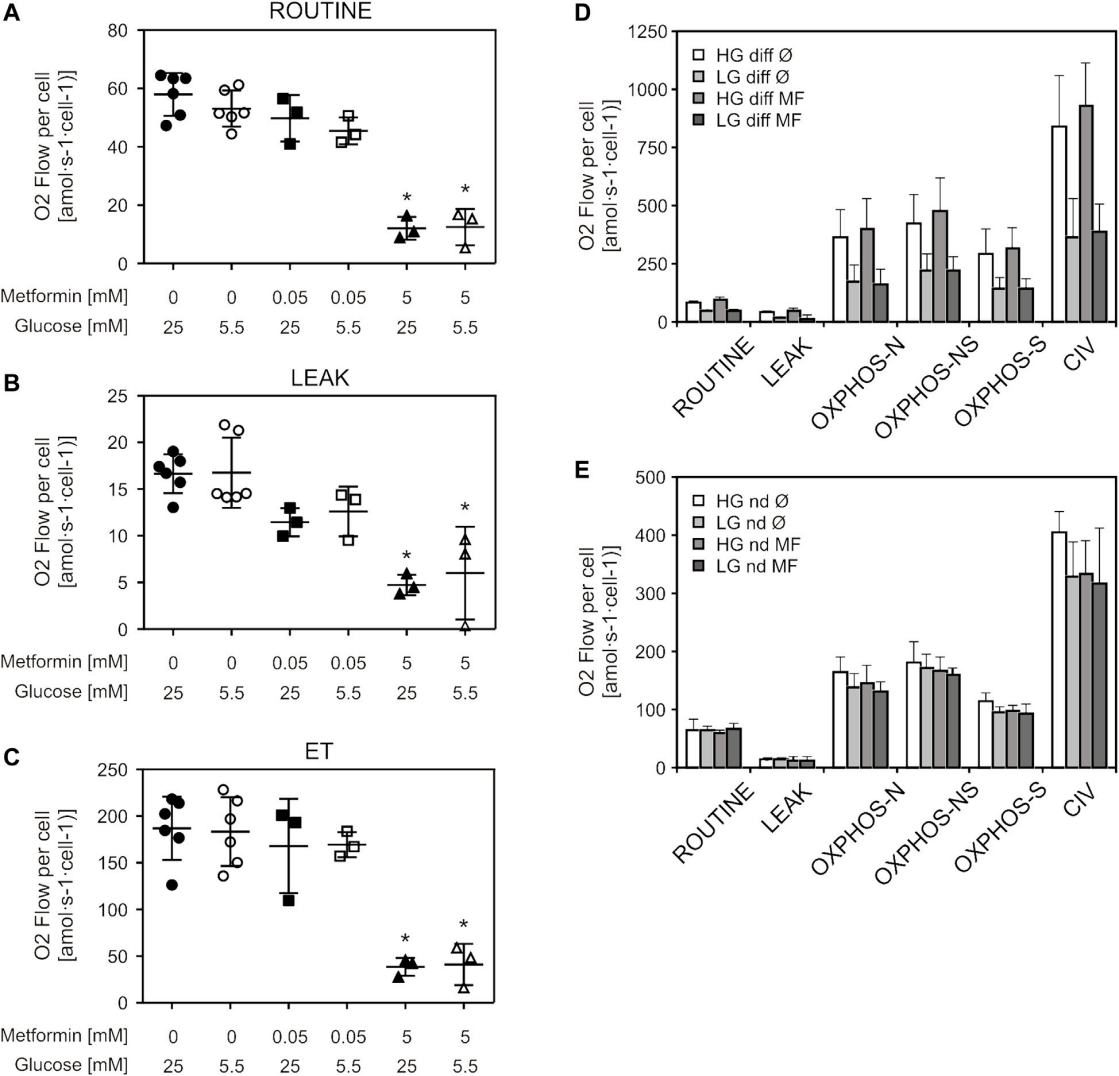
Therapeutic metformin concentrations did not alter mitochondrial respiration of living cells or long-term treated permeabilized cells. C2C12 cells were treated with 50 µM or 5 mM metformin for 24 h and respiration of living (intact) cells was measured by high-resolution respirometry. Respiration rates of ROUTINE **(A)**, LEAK **(B)** and ET **(C)** respiratory states, corrected for ROX, are presented as scatter plots, with mean ± SD (number of independent experiments *n* = 3). Differentiated **(D)** and undifferentiated **(E)** C2C12 cells were treated with 50 µM metformin for 5 days, and respiration of permeabilized cells was measured by high-resolution respirometry. Results from three independent experiments are presented as mean ± SD, *p < 0.05. HG—high glucose, LG—low glucose, MF—metformin, Ø—untreated control, diff—differentiated cells, nd—undifferentiated cells, ET—electron transfer capacity, OXPHOS-N—NADH-linked oxidative phosphorylation capacity, OXPHOS-NS—NADH and succinate-linked oxidative phosphorylation capacity, OXPHOS-S—succinate-linked oxidative phosphorylation capacity.

We wanted to further explore the effect of metformin on mitochondrial respiration by using a different protocol for high-resolution respirometry (respiration of living cells) as well as different treatment durations—a short treatment (adding metformin directly to the instrument chamber prior to measurement) and a 5-day treatment of undifferentiated and differentiated cells. Differentiated C2C12 cells were added in order to explore possible differences in response to metformin treatment between undifferentiated and differentiated cells, as differentiation has been shown to affect energy metabolism in C2C12 cells (Siengdee et al., 2015). Measuring respiration of intact cells, we observed decreased respiration in cells treated with 5 mM (but not 50 µM) metformin, in all three measured respiratory states (ROUTINE, LEAK, ET) (Figures 3A–C). Similarly, NADH-linked OXPHOS capacity decreased only in cells exposed to short-term 5 mM metformin treatment (Supplementary Figure S1C). We observed no changes in differentiated and undifferentiated cells treated with 50 µM metformin for 5 days, in any of the respiratory states or either cell culture medium (Figures 3D,E). Citrate synthase activity, as a measure of mitochondrial content, showed no difference between either untreated and cells treated with metformin for 5 days, or cells cultured in LG and HG medium [HG: Ø = 0.033 ± 0.026; MF = 0.035 ± 0.029; LG: Ø = 0.05 ± 0.01; MF = 0.053 ± 0.013 (IU/10^6^ cells)] Respiratory data normalized to citrate synthase activity also showed no difference between groups (data not shown).

**FIGURE 4:**
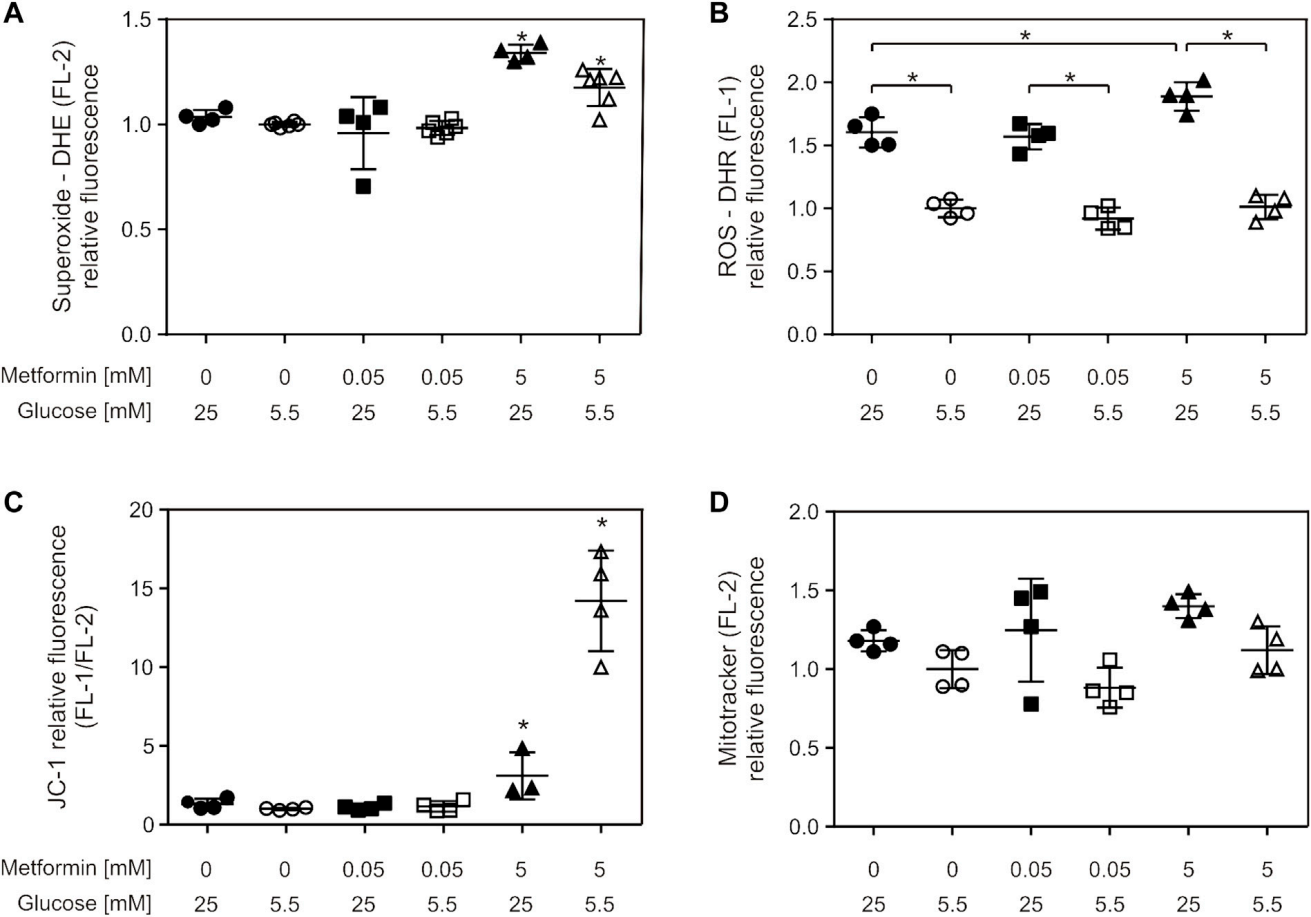
Suprapharmacological, but not therapeutic, metformin concentration caused increased total ROS and superoxide production and mitochondrial membrane depolarization. C2C12 cells were treated with 50 µM or 5 mM metformin for 24 h, and superoxide **(A)**, ROS **(B)**, mitochondrial membrane potential **(C)** and mitochondrial content **(D)** were measured by flow cytometry using appropriate fluorophores. All the data points from two independent experiments done in duplicate are presented as scatter plots, with mean ± SD, *p < 0.05.

Superoxide production was increased by 5 mM metformin treatment in both cell media, which was more pronounced for HG (34%) compared to LG—cultured cells (18%) (Figure 4A). Furthermore, 5 mM metformin treatment also increased ROS production in HG medium (Figure 4B). Total ROS production was elevated in HG medium, irrespective of metformin treatment, when compared to LG medium-grown cells (Figure 4B). 5 mM metformin caused depolarization of inner mitochondrial membrane in both media, the effect being more pronounced in LG medium-grown cells (mean FL1/FL2 for HG = 3.1, LG = 14.2) (Figure 4C). Total mitochondrial content did not change in any of the studied conditions (Figure 4D). It should be noted that mitochondrial membrane depolarization is also evident at earlier time points (starting from 6 h), whereas the values of other parameters remained unchanged (data not shown).

**FIGURE 5:**
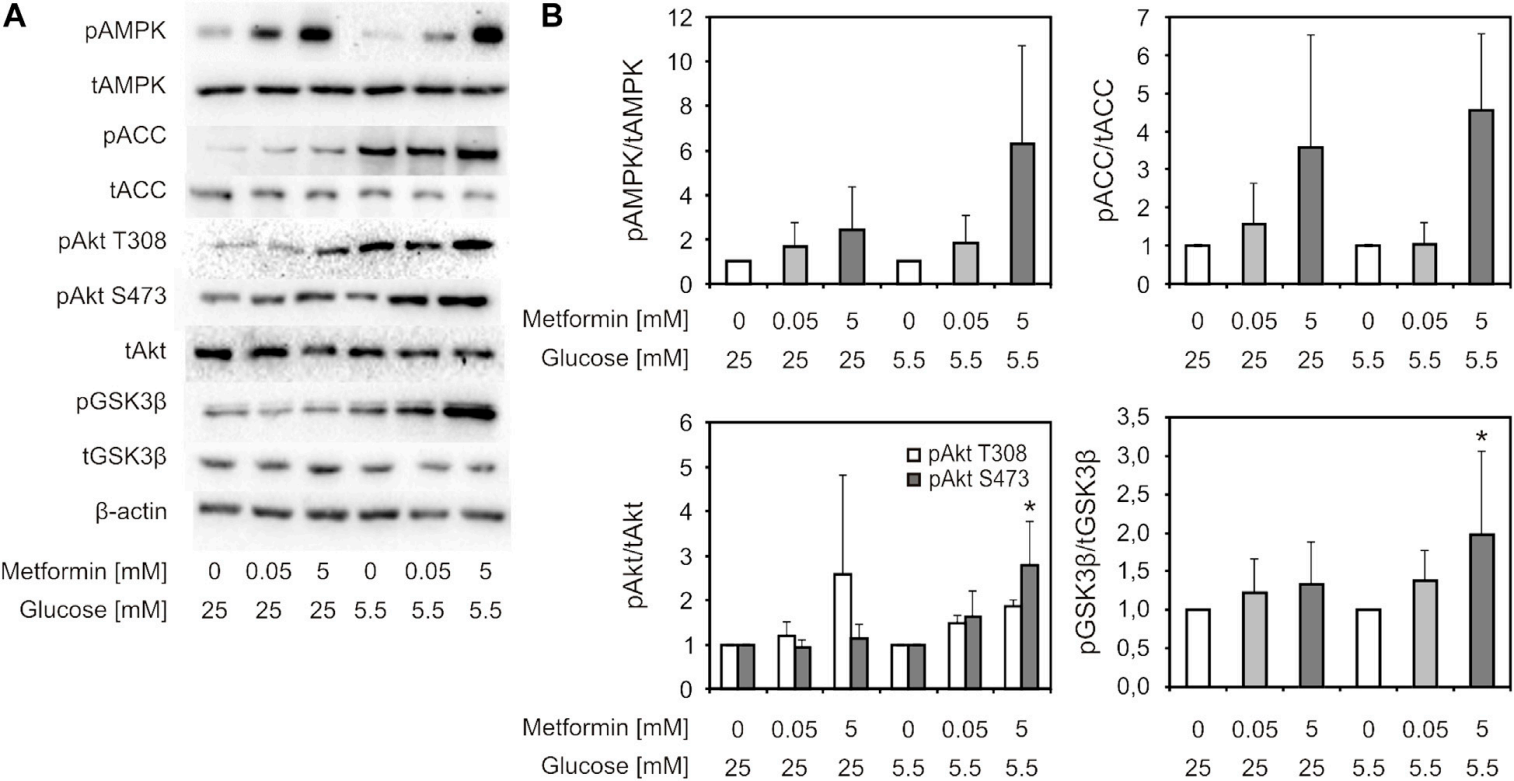
AMPK and Akt signaling pathways were activated by suprapharmacological, but not therapeutic metformin concentration. C2C12 cells were treated with 50 µM or 5 mM metformin for 24 h. Representative immunoblot analysis of proteins of interest—phosphorylated and total forms (pAMPK Thr172, AMPK, pAkt Ser473, pAkt Thr308, Akt, pGSK3β Ser9, GSK3β, pACC Ser79, ACC, β-actin) of one of three indipendent experiments with similar results **(A)**. Densitometry data **(B)** is presented as fold change ±SD, compared to untreated control set to 1. Protein levels were normalised to β-actin, and phosphorylated protein levels were expressed relative to total protein level. *p < 0.05 compared to control, number of independent experiments *n* ≥ 3.

Activity of PI3K/Akt and AMPK signaling pathways was studied using western blot (Figure 5A). In cells treated with 5 mM, but not 50 µM metformin, a trend of increase in AMPK (Thr172) and its downstream substrate ACC phosphorylation level was observed in both HG and LG medium (2-5 fold and 2-7 fold, respectively), which implies activation of this signaling pathway (Figure 5B, upper panels). The change was more pronounced in LG medium. The same trend was also observed for phosphorylated Akt, including both phosphorylation sites (1.5-5 fold and 1.5–3.5 fold increase for Thr308 and Ser473, respectively), and phosphorylation of GSK3β (downstream substrate of Akt) (1.3–3.5 fold increase), reaching statistical significance for pAkt S473 and GSK3β in LG medium-cultured cells (Figure 5B, lower panels).

## Discussion

We found that low concentrations of metformin, representative of the therapeutic concentration *in vivo*, have a different effect in muscle cells than the vastly studied millimolar concentrations. Micromolar metformin treatment failed to induce Complex I inhibition or AMPK activation. Additionally, glucose availability in cell medium slightly changed the cells’ response to higher metformin concentrations. Metformin had a more pronounced effect on Complex I inhibition, mitochondrial membrane depolarization and AMPK activation in cells cultured in low glucose medium, but cells cultured in high glucose medium were more prone to induction of ROS production.

Data on how metformin affects cell viability is scarce, and the majority of studies that explore mechanisms of metformin action fail to report if the investigated concentrations impact cell viability, even if these concentrations are quite high. Metformin has been shown to cause cell death in tumor cells (Wang Y. et al., 2018; Zhao et al., 2018; Teh et al., 2019), and some non-tumor cell lines (Wang X. et al., 2018; Deng and Ma, 2018), but also to counteract cytotoxic effects of other substances, like lipids and high glucose (Kim et al., 2010; Jadhav et al., 2013; Langer et al., 2016; Geng et al., 2020). According to our results, 24 h treatment with millimolar metformin concentrations caused a decrease in cell viability, which is another fact pointing to the questionable relevance of using high concentrations for exploring possible mechanisms behind metformin’s therapeutic effects. Complex I inhibition has been proposed as one of the mechanism behind the antineoplastic effect of metformin, and it has been shown that tumor cells are more prone to metformin-induced cell death when glucose-starved (Wheaton et al., 2014; Samuel et al., 2017). As metformin has been shown to decrease ATP production (El-Mir et al., 2000; Griss et al., 2015), low glucose concentration in cell medium would render the cells less capable to compensate for this, and thus more sensitive to metformin-induced cell death. Considering this, we hypothesized that C2C12 cells grown in LG medium are more sensitive to cytotoxic effects of metformin. Nevertheless, a more pronounced decrease in cell viability in cells grown in LG medium was observed only in MTT assay.

In the majority of *in vitro* studies that showed metformin inhibiting mitochondrial Complex I (using respirometry or isolated Complex I activity measurement) suprapharmacological, millimolar concentrations were applied (El-Mir et al., 2000; Owen et al., 2000; Carvalho et al., 2008; Bridges et al., 2014; Pecinova et al., 2017), which are significantly higher than plasma concentrations in patients on oral therapy (0.4–30 µM) (Christensen et al., 2011) and experimental animals (Wilcock et al., 1991). The rationale for using high metformin concentrations *in vitro* is the assumed accumulation of positively charged metformin molecules in the mitochondrial matrix, which could, due to mitochondrial membrane potential, occur in a therapeutic setting (Owen et al., 2000; Bridges et al., 2014). According to metformin pharmacodynamics and the fact that, to this day a transporter on the inner mitochondrial membrane that would allow this hydrophilic molecule to enter the matrix has not been identified, this hypothesis seems unlikely (Glossmann and Lutz, 2019). Also, by measuring radio-labelled metformin in different cellular fractions (Detaille et al., 2002; Wang et al., 2019) it was not possible to detect a significant enough accumulation in mitochondria. Some recent studies in hepatocytes and cardiomyocytes reveal the biphasic nature of metformin action, with high concentrations inhibiting mitochondrial respiration, and low concentrations exerting different effects or even increasing mitochondrial respiratory function (Alshawi and Agius, 2019; Wang et al., 2019; Emelyanova et al., 2021). As for *in vivo* studies, metformin therapy in experimental animals and humans has been shown by some to decrease mitochondrial respiration (Wessels et al., 2014), while others claim that metformin had no effect on mitochondrial respiration of skeletal muscle measured *ex vivo* (Owen et al., 2000; Larsen et al., 2012).

One of the hallmark papers on the topic (El-Mir et al., 2000) claims that metformin (in millimolar concentrations) caused inhibition of Complex I respiratory activity in intact liver cells, but not in isolated mitochondria and permeabilized cells, explaining this difference by the activation of a yet unknown signaling pathway causing Complex I inhibition. Another paper published the same year (Owen et al., 2000) showed that even micromolar metformin treatment (24 and 60 h) of rat hepatoma cells caused a decrease in Complex I-linked respiration. Respiration of isolated mitochondria and sub-mitochondrial particles (SMPs) was also inhibited by short treatments of millimolar metformin, with SMPs requiring much higher concentrations in order to cause inhibition. This difference in IC_50_ values is explained by accumulation of metformin inside the mitochondria. Considering this hypothesis, our experimental setting should allow for metformin to accumulate and reach concentrations sufficient for Complex I inhibition. However, this was not observed, as mitochondrial respiration was unaltered after 24-h and even 5-day 50 µM metformin treatment. One commentary article (Fontaine, 2014) indicates that the different interpretation of similar results in these two papers is due to different measured respiratory states—while one group performed all measurements in OXPHOS state (state 3) (Owen et al., 2000), the other measured respiration in LEAK state (state 4) (El-Mir et al., 2000), in experiments where they did not observe inhibition of respiration in either permeabilized cells or isolated mitochondria. The paper states that, according to previous work on biguanides, metformin-induced inhibition of respiration is observed in OXPHOS state, but is not as evident in LEAK or ET. The fact that O_2_ flux remained unaltered by metformin in ET (uncoupled) state is explained by the previously mentioned assumption that metformin accumulates inside polarized mitochondria, and that dissipation of membrane potential by an uncoupler would attenuate metformin accumulation and subsequent Complex I inhibition. To the best of our knowledge, no explanation for why metformin would not exert its effects in LEAK state was offered so far. According to our data on respiration of permeabilized cells, 5 mM metformin decreased OXPHOS but not LEAK respiration (ET was not measured). On the other hand, 5 mM metformin caused a decrease in all three measured respiratory states in living cells (ROUTINE, LEAK, ET), contradictory to the previously discussed hypothesis. It is important to note that these LEAK states are not identical—in permeabilized cells LEAK state is measured in absence of ADP [as in the discussed publication (El-Mir et al., 2000)], and in living cells by adding the ATP-synthase inhibitor oligomycin. This underlines the significance of standardization of protocols for respirometry, as using different experimental conditions and nomenclature makes it difficult to reproduce and compare published data.

Metformin has been shown to increase ROS production in isolated mitochondria, when using extremely high concentrations (>25 mM) (Bridges et al., 2014; Pecinova et al., 2017). On the other hand, metformin has been shown to decrease ROS production (Emelyanova et al., 2021) or return it to control levels in animal and cell models of diabetes and ischemia (Kane et al., 2010; Mohsin et al., 2019; Liu et al., 2020). Our results show an increase in ROS and superoxide production caused by 5 mM metformin, which was only present in HG medium for total ROS and more pronounced in said medium for superoxide. Also, total ROS production was higher in HG when compared to LG cells, independently of metformin. It is known that hyperglycemia causes oxidative stress in many tissues (Fiorentino et al., 2013; Luc et al., 2019), resulting in diabetes complications, but so-called glucotoxicity has not been extensively studied in skeletal muscle. The link between Complex I inhibition and ROS production (both induced by 5 mM metformin in our experiments) is not clear-cut, as Complex I inhibition can result in both an increase and a decrease in ROS production, depending on the conditions (Scialò et al., 2017). While not in accordance with some of the previously published data (Wheaton et al., 2014; Wang et al., 2019), our results show mitochondrial membrane depolarization by high concentrations of metformin. This is to be expected as a consequence of Complex I inhibition, as previously shown for metformin (Carvalho et al., 2008), as well as rotenone, a typical Complex I inhibitor (Xiong et al., 2013; Hong et al., 2014; Fortalezas et al., 2018). This result, along with the decrease of respiration in uncoupled ET state, contradicts the statement that metformin-induced Complex I inhibition is specific for polarised mitochondria. Metformin has been previously shown to alter processes of mitochondrial dynamics (Terada et al., 2002; Zong et al., 2002; Krogh et al., 2016; Wang et al., 2019; Crocker et al., 2020). However, our data from flow cytometry and citrate synthase activity show that metformin did not cause changes in mitochondrial content, in accordance to some of the previously published results (Wessels et al., 2014).

Activation of AMPK is the most well studied and universally accepted mechanism of metformin action, and it has been shown in skeletal muscle in numerous *in vivo* (Musi et al., 2002; Sajan et al., 2010), as well as *in vitro* studies using millimolar metformin treatment (Kalender et al., 2010; Sajan et al., 2010; Chen et al., 2011; Ouyang et al., 2011). Only a small number of *in vitro* studies on hepatocytes obtained similar results with therapeutic metformin concentrations (Zhou et al., 2001; Wang et al., 2019). Metformin in micromolar concentrations failed to induce AMPK activation in C2C12 cells (Bruckbauer and Zemel, 2013). Insulin signaling pathway activation by metformin was observed in some studies (Fantus and Brosseau, 1986; Kumar and Dey, 2002; Gunton et al., 2003), but not in others (Kim et al., 2002; Miller et al., 2013). Metformin was mostly studied in context of reversing the effects of insulin resistance on the insulin signaling pathway (Wu et al., 2015; Zhou et al., 2016; Zabielski et al., 2017; Bradley et al., 2019). These two pathways respond to opposite cues - AMPK is activated when nutrients are deficient and PI3K/Akt when they are abundant. On the other hand, they lead to some converging effects, especially in skeletal muscle, where both induce glucose uptake and GLUT4 exocytosis (Towler and Hardie, 2007). These pathways are typically reciprocally regulated by inhibiting one-another (Hahn-Windgassen et al., 2005; Towler and Hardie, 2007; Salminen et al., 2016), but there is a growing body of evidence suggesting this interaction is more complex than previously assumed, with many examples of AMPK activating the insulin signaling pathway (Bertrand et al., 2006; Leclerc et al., 2010; Tao et al., 2010). How metformin fits into this interaction is not well studied—in cancer cells metformin has been shown to cause cell death by activating AMPK and thus inhibiting Akt (Wang Y. et al., 2018; Lu et al., 2019; Chen et al., 2021), but data in non-tumor cells is scarce (Kovacic et al., 2003). Studying the effects of metformin treatment on these two pathways using pharmacological or genetic modulation of their activity presents a promising future research perspective. By measuring protein phosphorylation levels, we observed that AMPK and PI3K/Akt pathways were both activated only by millimolar metformin concentrations, more so in LG medium. Both pathways having a stronger response to metformin activation in LG medium can be explained by low glucose concentration inducing AMPK activation independently of metformin (Salt et al., 1998), and high glucose concentrations (which lead to insulin resistance) inhibiting the activity of the insulin signaling pathway (Huang et al., 2002; Samuel and Shulman, 2016). According to some authors, by inhibiting mitochondrial respiratory function, metformin causes a decrease in ATP production and a subsequent change in cell energy status (ADP/ATP and AMP/ATP ratios), which leads to AMPK activation (Stephenne et al., 2011), while others claim that AMPK activation by metformin is independent of Complex I inhibition (Ouyang et al., 2011). Our results show that millimolar metformin concentrations caused both Complex I inhibition and AMPK activation, while therapeutic concentrations caused none of these effects. The overall mechanisms behind metformin-induced AMPK activation in skeletal muscle are still insufficiently elucidated and are currently being investigated in our lab.

Effects of therapeutic metformin concentrations on energy metabolism have recently been extensively studied in liver cells—changes in energy and redox status, allosteric regulation of metabolic pathway enzymes (Gui et al., 2017; Cameron et al., 2018; Alshawi and Agius, 2019) and inhibition of glycerol-3-phosphate dehydrogenase (Madiraju et al., 2014, 2018; Thakur et al., 2018) may have replaced Complex I inhibition as a central mechanism of metformin action. These mechanisms have not been studied in skeletal muscle, as it has been sidetracked and considered not as important for the therapeutic effects of metformin. As a metabolically highly active tissue that plays an important role in glycemia regulation, and with peripheral insulin resistance arising before hepatic (Hales, 1994), studying the effects of metformin on skeletal muscle is important for diabetes treatment and prevention strategies (eg., pharmacological therapy in prediabetes).

In conclusion, our results show that therapeutic metformin concentrations lead to different effects compared to widely used millimolar concentrations—they did not cause the previously described effects on mitochondrial respiration and AMPK signaling. Even though suprapharmacological metformin concentrations were shown to cause Complex I inhibition and AMPK activation in skeletal muscle cells *in vitro*, therapeutic concentrations did not induce such an effect. This raises the question if these mechanisms are relevant for therapeutic effects of metformin in skeletal muscle.

## Data Availability

The raw data supporting the conclusions of this article will be made available by the authors, without undue reservation.

## Supplementary Material

Supplementary Figure S1 C2C12 cells were treated with metformin (50 and 5 mM) by adding the treatment directly to the instrument chamber and mitochondrial respiration of permeabilized cells was measured by high-resolution respirometry. Results for ROUTINE **(A)**, LEAK **(B)**, OXPHOS-N **(C)** and OXPHOS-S **(D)** respiratory states, all corrected for ROX, from 3 to 5 independent experiments are presented as scatter plots, with mean ± SD, **p*<0.05. OXPHOS-N—NADH-linked oxidative phosphorylation capacity, OXPHOS-S—succinate-linked oxidative phosphorylation capacity.
