# Supplementary figures and images for: Therapeutic vs. Suprapharmacological Metformin Concentrations: Different Effects on Energy Metabolism and Mitochondrial Function in Skeletal Muscle Cells in vitro
Source: Front Pharmacol. 2022 Jul 6;13:930308. doi: 10.3389/fphar.2022.930308 (PMC9299382; doi:10.3389/fphar.2022.930308)

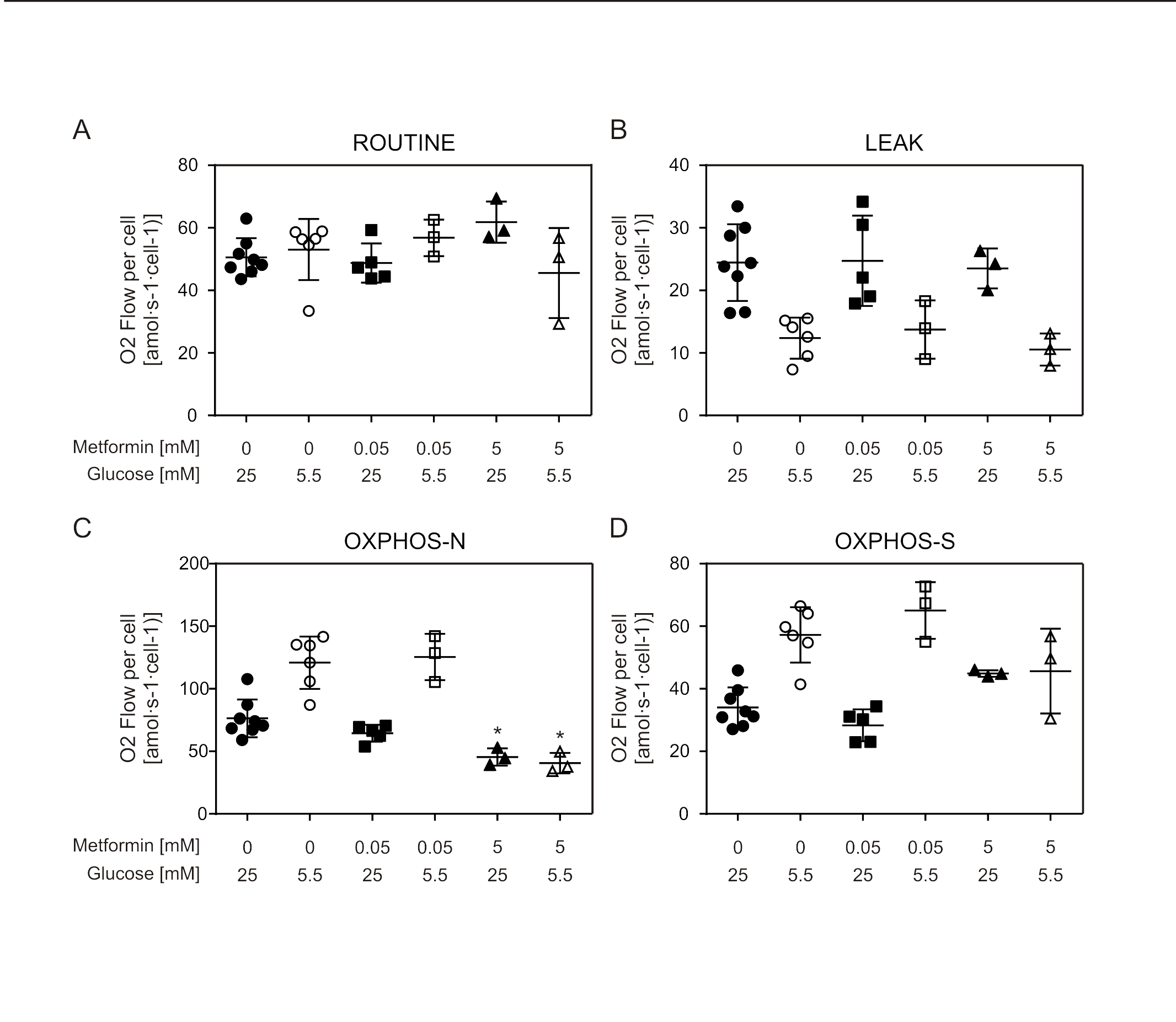

Supplement: Supplementary file 1 [file Image1.TIF]
